# Skeletal muscle metabolism on whole-body positron emission tomography during pitching

**DOI:** 10.1186/s12970-021-00418-4

**Published:** 2021-03-06

**Authors:** Yasushi Takata, Junsuke Nakase, Anri Inaki, Takafumi Mochizuki, Kengo Shimozaki, Kazuki Asai, Seigo Kinuya, Hiroyuki Tsuchiya

**Affiliations:** 1 Department of Orthopaedic Surgery, Graduate School of Medical Science, Kanazawa University, 13-1 Takaramachi, Kanazawa, 920-0934 Japan; 2 Department of Orthopaedic Surgery, National Hospital Organization, Kanazawa Medical Center, Kanazawa, Japan; 3 Department of Nuclear Medicine/Biotracer Medicine, Graduate School of Medical Science, Kanazawa University, Kanazawa, Japan; 4 Kanazawa Advanced Medical Center, Kanazawa, Japan

**Keywords:** Baseball, Glucose metabolism, Muscle strengthening, Shoulder injuries, Injury prevention, Performance enhancement, Muscles metabolism

## Abstract

**Background:**

Electromyography (EMG) has been used for evaluating skeletal muscle activity during pitching. However, it is difficult to observe the influence of movement on skeletal muscle activity in deep-lying regions of the trunk and extremities using EMG. An alternative method that may be used is the measurement of glucose metabolism of skeletal muscle using positron emission tomography-computed tomography (PET-CT). This technique is a reliable measure of muscle metabolism, demonstrating a high correlation with the intensity of muscle activity. This study aimed to evaluate whole-body skeletal muscle metabolism during pitching using PET-CT.

**Methods:**

Ten uninjured, skilled, adult pitchers, who were active at college or professional level, threw 40 baseballs at maximal effort before an intravenous injection of 37 MBq of ^18^F-fluorodeoxyglucose (FDG). Subsequently, additional 40 balls were pitched. PET-CT images were obtained 50 min after FDG injection, and regions of interest were defined within 72 muscles. The standardized uptake value (SUV) of FDG by muscle tissue per unit volume was calculated, and the mean SUV of the pitchers was compared with that of a healthy adult control group who did not exercise before the measurements. Statistical analysis was performed using a t-test, and *P* < 0.05 was considered statistically significant.

**Results:**

Whole-body PET images showed a significant increase in glucose metabolism in the muscle groups of the fingers and toes in both the throwing and non-throwing sides. Additionally, asymmetric increases in glucose metabolism were observed in the muscles of the thigh.

**Conclusions:**

This is the first study to evaluate whole-body muscle metabolism during pitching using PET-CT. Our findings would be useful in determining the training required for pitchers, and can be further applied to other sporting activities that involve throwing.

## Background

The baseball pitching motion is one of the fastest human motions [1]. Thus, shoulder and elbow pain related to the pitching motion is highly prevalent (approximately 46–57% of pitchers) [2]. Several studies have been conducted on the prevention of injuries during pitching and the improvement of performance [1–14]. Many of these studies have evaluated motion analysis and skeletal muscle activity using electromyography (EMG) [1, 2, 4–12, 14]. Skeletal muscle activity during pitching has been identified for each body part using EMG [4–12, 14]. Until now, EMG has been the gold standard method in the evaluation of skeletal muscle activity [3]. However, this study focused on positron emission tomography (PET) as a method of evaluating whole-body skeletal muscle metabolism. With PET, it is possible to evaluate whole-body skeletal muscle metabolism in a one-time examination [15, 16]. Understanding the patterns of whole-body skeletal muscle metabolism in baseball pitching is important not only to define the role of each body part but also to assist in performance enhancement and decrease potential injury [4]. The evidence provided in one study suggests that increased pitching workload may be associated with an increased risk of pain, injury, and arm fatigue in pitchers [17]. Knowledge of the whole-body skeletal muscle metabolism during pitching may facilitate a consideration of other training methods to obtain the necessary muscle strength for pitching. Consequently, it may contribute to reducing overuse injuries and improving performance.

This study aimed to evaluate whole-body skeletal muscle metabolism during a pitching exercise using PET-computed tomography (PET-CT). Based on previous EMG studies and the common occurrence of shoulder injuries among pitchers, it was hypothesized that skeletal muscle metabolism increases in the muscles around the shoulder during pitching.

## Methods

### Participants

Voluntary applicants were recruited to participate in this study. Those with relevant medical histories, complications, complaints such as pain, and those who did not provide their written consent were excluded. The purpose and potential risks of this study were explained to each participant, who provided written informed consent to participate. The study design was approved by the Ethics Committee of Kanazawa University Hospital (2251–1) and Kanazawa Advanced Medical Center (070209). All human experiments in this study followed the guidelines of the Declaration of Helsinki.

Ten uninjured, skilled, healthy adult male pitchers, who were active at college or professional level with no restriction that would limit maximal effort pitching, participated in this study as the pitcher group. Their physical characteristics (mean ± standard deviation [SD]) were as follows: age, 21.5 ± 3.7 years; height, 175.9 ± 3.4 cm; weight, 74.7 ± 5.2 kg; body mass index, 24.2 ± 1.8 kg/m^2^. Ten healthy adult men who do not exercise and only perform daily life activities served as controls and underwent PET scans to detect the accumulation of ^18^F-fluorodeoxyglucose (FDG). Their physical characteristics (mean ± SD) were as follows: age, 24.1 ± 4.1 years; height, 174.1 ± 4.9 cm; weight, 78.8 ± 7.4 kg; body mass index, 25.95 ± 1.9 kg/m^2^. Based on a previous study [15], it was assumed that there would be a two-fold increase in muscle glucose uptake after exercise. None of the participants was on any medication and were all considered healthy based on their medical history and physical examination.

### Methodology

All participants refrained from eating and drinking for at least 6 h before the FDG-PET assessment, and strenuous physical activity was avoided for at least 1 day before the experiment. The plasma glucose level of each participant was confirmed to be within the normal range (3.89–5.55 mmol/l) before an FDG injection was administered.

After performing sufficient warm-up exercises, the participants threw a total of 40 fast balls to a catcher at maximal effort, on the mound, for 20 min. Subsequently, 37 MBq of FDG was intravenously injected. Additional 40 fast balls were then pitched at maximal effort for another 20 min, followed by 25 min of rest in a sitting position. According to previous studies [15, 16], the influence of exercise is sufficiently reflected by performing the exercise twice (before and after FDG injection). The participants were verbally encouraged to generate maximal effort during pitching. The PET-CT images were obtained 50 min after FDG injection. The participants were subsequently placed in a supine position on a scanning bed that facilitated longitudinal placement into the gantry of the PET-CT system (Discovery PET/CT 690; GE Healthcare, Milwaukee, WI, USA). Scanning was performed with a 60-cm axial field of view and a transaxial resolution of 6.4 mm (full-width at half-maximum at the center field of view without a scattering medium). Before emission scanning, an unenhanced CT scan was performed for attenuation correction and anatomical orientation. Furthermore, emission scanning was performed in 3-dimensional (3D) mode 50 min after FDG administration, and the total emission time ranged from 39 to 42 min. Images were reconstructed using a 3D ordered-subset expectation-maximization algorithm with two iterations and 16 subsets. After reconstruction, a 6.4-mm FWHM Gaussian post-filter was applied.

Regions of interest (ROI) were manually segmented in 72 skeletal muscles (of the whole-body) from the basal neck to the foot. One experienced orthopedic specialist defined all ROIs using plain CT images. The standardized uptake value (SUV) was calculated by overlapping the defined ROI and fusion images. Large vessels were avoided in the course of outlining the muscle areas. The SUV was calculated to quantitatively examine the FDG uptake of the muscle tissue per unit volume according to the equation: SUV = {mean ROI count (counts per second [cps]/pixel) × calibration factor (cps/Bq)}/{injected dose (Bq)/body weight (g)}. The ROIs were defined for the right and left sides of the 72 skeletal muscles, and the mean SUV was calculated using the equation below:

$$ \mathrm{mean}\ \mathrm{SUV}=\mathrm{SUV}/\mathrm{muscle}\ \mathrm{area} $$

### Statistical analyses

All data are presented as means and SDs. In the determination of the sample size, the α value, power, and effect size of Cohen’s d were set at 0.05, 0.95, and 2.0, respectively, and a sample size of *n* = 10 was calculated for both the pitcher and control groups. The Shapiro-Wilk test was performed to ensure a normal distribution. The mean differences of the values were compared using the t-test, and the effect size was presented using Cohen’s d. SPSS for Windows ver. 23 (SPSS Inc., Chicago, IL, USA) was used for the analysis. Notably, the minimum significance level was set at *P* < 0.05.

## Results

**Fig. 1:**
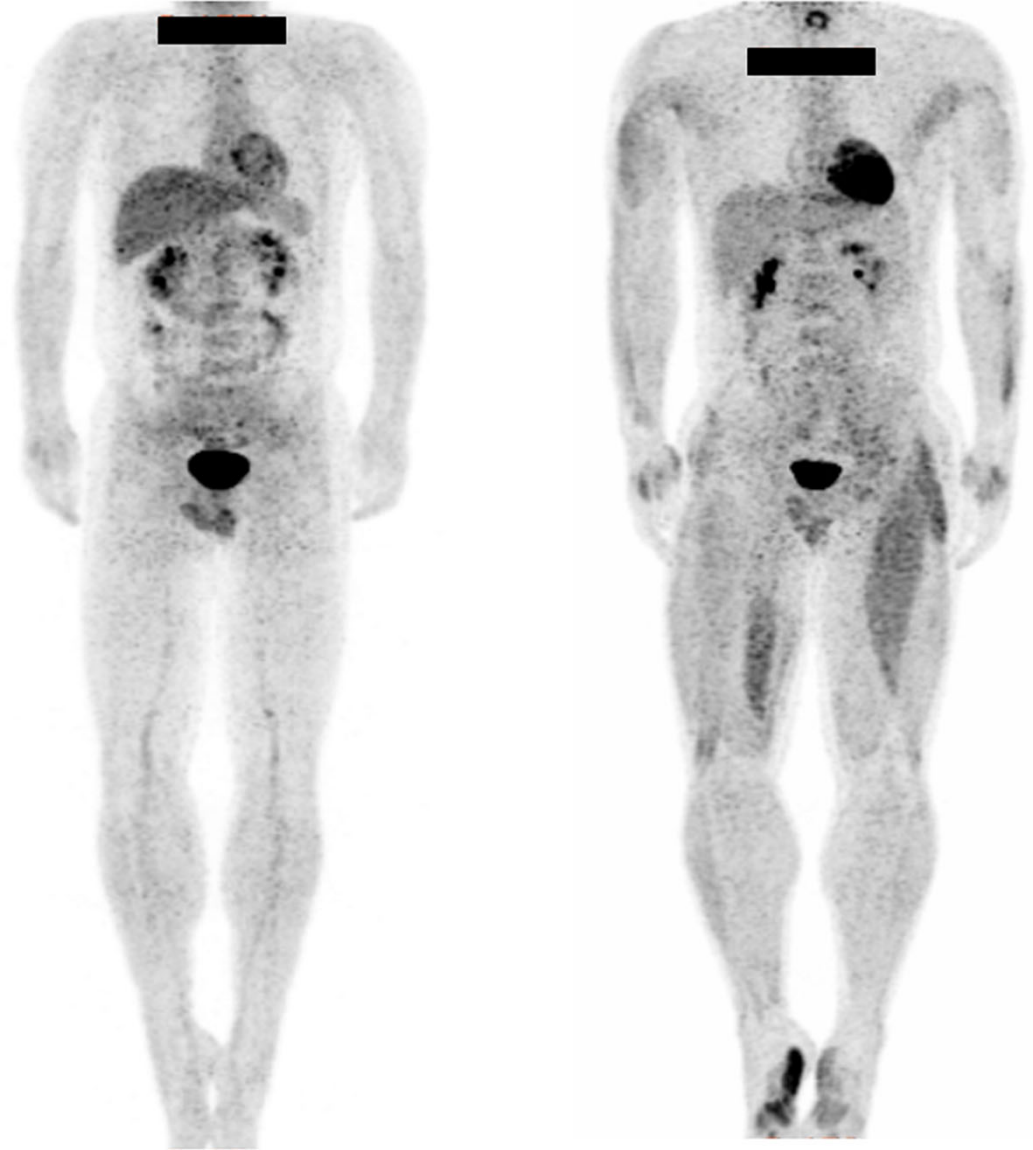
Representative whole-body positron emission tomography images of the control group (left side) and the pitcher group (right side)

Tables 1 and 2 show the mean SUVs measurements of the pitcher and control groups, and the 95% confidence intervals, effect sizes, and *P*-values of all muscles. The mean SUVs of the pitcher and control groups were normally distributed, allowing the use of a t-test. In the pitcher group, a significant increase in glucose metabolism was observed in 20 muscles of both the throwing (posterior part of the deltoid *P* = 0.017, biceps *P* = 0.021, brachialis *P* = 0.019, extensor digitorum *P* = 0.001, abductor hallucis brevis *P* = 0.007, adductor hallucis *P* = 0.007, tensor fasciae latae *P* = 0.02, biceps femoris *P* = 0.002, adductor complex *P* = 0.029, popliteus *P* = 0.01, anterior tibial *P* = 0.042, peroneus longus *P* = 0.013, peroneus brevis *P* = 0.000, abductor hallucis *P* = 0.016, plantar quadrate *P* = 0.028, flexor digitorum brevis *P* = 0.012, abductor digiti minimi *P* = 0.014, flexor hallucis brevis *P* = 0.001, abductor hallucis *P* = 0.017, interosseous *P* = 0.005) and non-throwing sides (posterior part of the deltoid *P* = 0.004, biceps *P* = 0.006, brachialis *P* = 0.011, extensor digitorum *P* = 0.000, abductor hallucis brevis *P* = 0.017, adductor hallucis *P* = 0.026, tensor fasciae latae *P* = 0.049, biceps femoris *P* = 0.006, adductor complex *P* = 0.017, popliteus *P* = 0.014, anterior tibial *P* = 0.038, peroneus longus *P* = 0.007, peroneus brevis *P* = 0.008, abductor hallucis *P* = 0.009, plantar quadrate *P* = 0.043, flexor digitorum brevis *P* = 0.015, abductor digiti minimi *P* = 0.01, flexor hallucis brevis *P* = 0.001, abductor hallucis *P* = 0.003, interosseous *P* = 0.012), 13 muscles of the throwing side (superior part of the trapezius *P* = 0.026, horizontal part of trapezius *P* = 0.024, teres major *P* = 0.017, latissimus dorsi *P* = 0.045, brachioradialis *P* = 0.038, lumbricalis *P* = 0.036, gluteus maximus *P* = 0.035, vastus intermedius *P* = 0.016, vastus medialis *P* = 0.011, semimembranosus *P* = 0.029, extensor hallucis longus *P* = 0.022, extensor digitorum longus *P* = 0.031, flexor hallucis longus *P* = 0.041) and 8 muscles of the non-throwing side (levator scapulae *P* = 0.049, forearm flexors *P* = 0.015, iliacus *P* = 0.005, gluteus minimus *P* = 0.039, sartorius *P* = 0.005, gracilis *P* = 0.005, semitendinosus *P* = 0.008, triceps surae *P* = 0.035). In summary, the results of the pitchers’whole-body PET images (Fig. 1) revealed significant increases in glucose metabolism in the muscle groups of the fingers and toes. In addition, in the lower limbs, asymmetric increases in glucose metabolism were observed in the thigh muscles. However, the rotator cuff or the trunk muscles did not exhibit increased glucose metabolism.

**Table 1:** SUVs for the upper extremity skeletal muscles between the pitcher and control groups

Muscles	Throwing side					Non-throwing side				
	SUV value		Confidence interval	Effect size	*P*- value	SUV value		Confidence interval	Effect size	*P*- value
	Pitching	Control				Pitching	Control			
Superior part of trapezius	0.70 ± 0.14	0.58 ± 0.08	[0.02, 0.24]	1.09	0.026^a^	0.64 ± 0.06	0.57 ± 0.09	[−0.01, 0.14]	0.85	0.074
Horizontal part of trapezius	0.74 ± 0.09	0.65 ± 0.08	[0.01, 0.17]	1.11	0.024^a^	0.74 ± 0.14	0.67 ± 0.08	[−0.03, 0.18]	0.67	0.153
Inferior part of trapezius	0.70 ± 0.07	0.73 ± 0.08	[−0.09, 0.05]	0.33	0.468	0.74 ± 0.13	0.69 ± 0.07	[−0.05, 0.15]	0.45	0.325
Levator scapulae	0.71 ± 0.18	0.58 ± 0.10	[−0.01, 0.27]	0.88	0.07	0.71 ± 0.15	0.58 ± 0.13	[0.00, 0.26]	0.94	0.049^a^
Rhomboids	0.65 ± 0.08	0.68 ± 0.11	[−0.11, 0.06]	0.27	0.554	0.67 ± 0.13	0.76 ± 0.09	[−0.19, 0.02]	0.76	0.106
Serratus anterior	0.76 ± 0.07	0.71 ± 0.08	[−0.06, 0.16]	0.46	0.316	0.74 ± 0.11	0.68 ± 0.10	[−0.04, 0.15]	0.56	0.23
Pectoralis minor	0.72 ± 0.09	0.62 ± 0.12	[−0.01, 0.19]	0.89	0.062	0.70 ± 0.14	0.68 ± 0.10	[−0.09, 0.14]	0.21	0.649
Subscapularis	0.76 ± 0.07	0.79 ± 0.08	[−0.10, 0.05]	0.32	0.481	0.80 ± 0.09	0.77 ± 0.09	[−0.06, 0.12]	0.3	0.509
Supraspinatus	0.75 ± 0.10	0.72 ± 0.14	[−0.08, 0.14]	0.25	0.579	0.76 ± 0.07	0.79 ± 0.09	[−0.10, 0.05]	0.33	0.465
Infraspinatus	0.81 ± 0.07	0.83 ± 0.09	[−0.10, 0.06]	0.22	0.626	0.86 ± 0.11	0.80 ± 0.11	[−0.04, 0.17]	0.56	0.227
Teres minor	0.88 ± 0.10	0.88 ± 0.19	[−0.14, 0.15]	0.05	0.906	0.88 ± 0.10	0.85 ± 0.14	[−0.09, 0.15]	0.25	0.589
Teres major	0.85 ± 0.22	0.63 ± 0.14	[0.04, 0.39]	1.17	0.017^a^	0.93 ± 0.49	0.65 ± 0.16	[−0.08, 0.64]	0.77	0.113
Anterior part of deltoid	0.60 ± 0.20	0.62 ± 0.12	[−0.18, 0.13]	0.14	0.758	0.68 ± 0.37	0.60 ± 0.10	[−0.18, 0.34]	0.3	0.515
Middle part of deltoid	0.66 ± 0.16	0.64 ± 0.11	[−0.11, 0.15]	0.12	0.787	0.66 ± 0.13	0.64 ± 0.09	[−0.09, 0.13]	0.18	0.69
Posterior part of deltoid	0.77 ± 0.10	0.65 ± 0.06	[0.04, 0.20]	1.46	0.017^a^	0.78 ± 0.11	0.63 ± 0.10	[0.06, 0.25]	1.49	0.004^a^
Latissimus dorsi	0.76 ± 0.29	0.55 ± 0.09	[0.01, 0.43]	1.02	0.045^a^	0.90 ± 0.49	0.57 ± 0.09	[−0.02, 0.68]	0.94	0.064
Pectoralis major	0.71 ± 0.22	0.56 ± 0.12	[−0.01, 0.32]	0.87	0.067	0.68 ± 0.37	0.58 ± 0.11	[−0.05, 0.26]	0.64	0.168
Coracobrachialis	0.97 ± 0.13	0.90 ± 0.12	[−0.05, 0.19]	0.56	0.229	0.66 ± 0.13	0.88 ± 0.14	[−0.15, 0.11]	0.13	0.769
Biceps	0.85 ± 0.33	0.57 ± 0.11	[0.05, 0.51]	1.13	0.021^a^	1.00 ± 0.36	0.59 ± 0.12	[0.15, 0.68]	1.54	0.006^a^
Brachialis	0.90 ± 0.23	0.67 ± 0.16	[0.04, 0.42]	1.16	0.019^a^	0.95 ± 0.31	0.63 ± 0.14	[0.09, 0.55]	1.32	0.011^a^
Triceps	0.74 ± 0.36	0.55 ± 0.10	[−0.07, 0.45]	0.72	0.137	0.60 ± 0.31	0.54 ± 0.12	[−0.15, 0.29]	0.29	0.519
Anconeus	0.77 ± 0.17	0.78 ± 0.14	[− 0.15, 0.14]	0.04	0.923	0.84 ± 0.21	0.74 ± 0.18	[−0.08, 0.29]	0.53	0.25
Pronator teres	0.77 ± 0.26	0.71 ± 0.19	[−0.15, 0.27]	0.26	0.562	0.82 ± 0.36	0.76 ± 0.18	[−0.20, 0.33]	0.22	0.631
Forearm flexors	0.96 ± 0.40	0.76 ± 0.08	[−0.08, 0.49]	0.71	0.144	1.04 ± 0.32	0.73 ± 0.10	[0.07, 0.54]	1.29	0.015^a^
Pronator quadratus	0.87 ± 0.53	0.72 ± 0.19	[−0.25, 0.53]	0.36	0.432	0.72 ± 0.16	0.72 ± 0.19	[−0.16, 0.17]	0.02	0.971
Brachioradialis	0.87 ± 0.30	0.64 ± 0.13	[0.01, 0.44]	1.00	0.038^a^	0.71 ± 0.28	0.54 ± 0.09	[−0.04, 0.37]	0.8	0.102
Extensor digitorum	1.67 ± 0.49	0.81 ± 0.15	[0.35, 1.09]	1.90	0.001^a^	1.96 ± 0.64	0.88 ± 0.17	[0.64, 1.51]	2.31	0.000^a^
Abductor hallucis brevis	1.67 ± 0.71	0.88 ± 0.30	[0.26, 1.32]	1.45	0.007^a^	1.30 ± 0.57	0.77 ± 0.14	[0.11, 0.94]	1.27	0.017^a^
Adductor hallucis	2.10 ± 0.88	1.13 ± 0.27	[0.33, 1.62]	1.50	0.007^a^	1.51 ± 0.54	1.03 ± 0.24	[0.07, 0.88]	1.13	0.026^a^
Abductor digiti minimi pedis	1.06 ± 0.64	0.94 ± 0.23	[−0.32, 0.57]	0.26	0.569	0.97 ± 0.29	0.86 ± 0.20	[−0.13, 0.35]	0.44	0.341
Lumbricalis	1.64 ± 0.77	1.02 ± 0.41	[0.05, 1.21]	1.02	0.036^a^	1.36 ± 0.67	0.97 ± 0.21	[−0.08, 0.85]	0.78	0.097

^a^Statistically significant

*SUV* Standardized uptake value

Values are presented as mean ± standard deviation

**Table 2:** SUVs for the trunk and lower extremity skeletal muscles between the pitcher and control groups

Muscles	Throwing side					Non-throwing side				
	SUV value		Confidence interval	Effect size	*P*-value	SUV value		Confidence interval	Effect size	*P*-value
	Pitching	Control				Pitching	Control			
Abdominal rectus	0.52 ± 0.09	0.57 ± 0.13	[− 0.16, 0.05]	0.66	0.313	0.50 ± 0.09	0.53 ± 0.11	[− 0.12, 0.07]	0.6	0.591
Abdominal external oblique	0.50 ± 0.05	0.46 ± 0.07	[−0.01, 0.10]	0.4	0.13	0.51 ± 0.07	0.48 ± 0.06	[−0.04, 0.09]	0.45	0.371
Abdominal internal oblique	0.64 ± 0.09	0.62 ± 0.09	[−0.07, 0.10]	0.07	0.676	0.67 ± 0.11	0.64 ± 0.10	[−0.07, 0.13]	0.88	0.572
Transverse abdominal	0.69 ± 0.12	0.69 ± 0.13	[−0.11, 0.11]	0.22	0.978	0.72 ± 0.17	0.60 ± 0.13	[−0.02, 0.26]	1.21	0.095
Greater psoas	0.85 ± 0.18	0.73 ± 0.13	[−0.08, 0.21]	0.37	0.359	0.91 ± 0.22	0.87 ± 0.12	[−0.13, 0.20]	0.38	0.622
Lumbar quadrate	0.66 ± 0.08	0.64 ± 0.14	[−0.08, 0.13]	0.52	0.641	0.68 ± 0.09	0.60 ± 0.11	[−0.01, 0.17]	0.52	0.089
Erector spinae	0.78 ± 0.13	0.76 ± 0.12	[−0.10, 0.13]	0.16	0.743	0.79 ± 0.10	0.74 ± 0.10	[−0.05, 0.14]	0.1	0.299
Iliacus	0.92 ± 0.10	0.98 ± 0.23	[−0.23, 0.11]	0.88	0.467	1.37 ± 0.36	0.95 ± 0.14	[0.16, 0.69]	1.52	0.005^a^
Gluteus maximus	0.75 ± 0.15	0.64 ± 0.06	[0.01, 0.22]	1.02	0.035^a^	0.80 ± 0.19	0.68 ± 0.10	[−0.03, 0.26]	1.05	0.1
Gluteus medius	0.76 ± 0.08	0.73 ± 0.10	[−0.06, 0.11]	0.17	0.566	0.86 ± 0.18	0.75 ± 0.05	[−0.02, 0.25]	0.73	0.092
Gluteus minimus	1.17 ± 0.57	0.98 ± 0.13	[−0.20, 0.58]	0.47	0.33	1.68 ± 1.00	0.91 ± 0.18	[0.05, 1.49]	0.9	0.039^a^
Piriformis	0.93 ± 0.16	0.93 ± 0.16	[−0.15, 0.15]	0.59	0.977	1.11 ± 0.27	0.97 ± 0.19	[−0.09, 0.35]	0.05	0.22
Internal obturator	0.93 ± 0.19	1.20 ± 0.37	[−0.54, 0.02]	1.45	0.062	0.99 ± 0.21	1.04 ± 0.37	[−0.34, 0.23]	0.5	0.712
Obturator externus	0.95 ± 0.17	1.00 ± 0.24	[−0.24, 0.16]	0.86	0.677	1.12 ± 0.33	1.02 ± 0.19	[−0.15, 0.35]	0.12	0.414
Tensor fasciae latae	0.60 ± 0.19	0.43 ± 0.08	[0.03, 0.31]	1.15	0.020^a^	0.89 ± 0.61	0.45 ± 0.05	[0.00, 0.88]	0.94	0.049^a^
Rectus femoris	0.67 ± 0.36	0.49 ± 0.08	[−0.06, 0.43]	0.67	0.13	0.90 ± 0.63	0.49 ± 0.08	[−0.05, 0.86]	0.78	0.074
Vastus lateralis	0.64 ± 0.12	0.55 ± 0.10	[−0.01, 0.19]	0.66	0.088	0.60 ± 0.15	0.55 ± 0.10	[−0.08, 0.17]	0.42	0.424
vastus intermedius	0.80 ± 0.12	0.64 ± 0.15	[0.03, 0.29]	1.51	0.016^a^	0.76 ± 0.11	0.69 ± 0.10	[−0.03, 0.17]	0.85	0.15
vastus medialis	0.68 ± 0.13	0.55 ± 0.06	[0.03, 0.23]	1.21	0.011^a^	0.62 ± 0.14	0.55 ± 0.06	[−0.03, 0.17]	0.73	0.14
Sartorius	0.66 ± 0.31	0.54 ± 0.10	[−0.10, 0.34]	0.43	0.264	0.92 ± 0.34	0.52 ± 0.08	[0.15, 0.64]	1.4	0.005^a^
Gracilis	0.58 ± 0.20	0.43 ± 0.10	[0.00, 0.30]	0.58	0.052	0.59 ± 0.16	0.40 ± 0.10	[0.07, 0.31]	0.98	0.005^a^
Semimembranosus	0.78 ± 0.32	0.52 ± 0.07	[0.03, 0.50]	0.92	0.029^a^	0.70 ± 0.21	0.55 ± 0.07	[0.00, 0.29]	0.84	0.051
Semitendinosus	0.88 ± 0.59	0.46 ± 0.10	[0.00, 0.84]	0.85	0.051	0.64 ± 0.15	0.48 ± 0.08	[0.05, 0.27]	1.44	0.008^a^
Biceps femoris	0.67 ± 0.11	0.53 ± 0.06	[0.06, 0.23]	1.77	0.002^a^	0.68 ± 0.12	0.54 ± 0.08	[0.05, 0.24]	1.59	0.006^a^
Adductor complex	0.74 ± 0.09	0.66 ± 0.07	[0.01, 0.16]	1.24	0.029^a^	0.75 ± 0.08	0.65 ± 0.09	[0.02, 0.17]	1.85	0.017^a^
Popliteus	0.98 ± 0.19	0.77 ± 0.11	[0.06, 0.36]	1.18	0.010^a^	0.97 ± 0.17	0.80 ± 0.12	[0.04, 0.32]	0.86	0.014^a^
Anterior tibial	1.18 ± 0.50	0.79 ± 0.17	[0.02, 0.76]	0.89	0.042^a^	1.28 ± 0.68	0.75 ± 0.20	[0.03, 1.03]	0.93	0.038^a^
Extensor hallucis longus	1.11 ± 0.32	0.81 ± 0.19	[0.05, 0.55]	1.05	0.022^a^	1.07 ± 0.33	0.88 ± 0.23	[−0.07, 0.46]	0.58	0.143
Extensor digitorum longus	0.98 ± 0.24	0.77 ± 0.13	[0.02, 0.39]	1.01	0.031^a^	1.06 ± 0.31	0.82 ± 0.17	[0.00, 0.47]	1.23	0.051
Peroneus longus	1.03 ± 0.39	0.66 ± 0.09	[0.10, 0.66]	1.23	0.013^a^	1.07 ± 0.38	0.67 ± 0.15	[0.12, 0.69]	1.09	0.007^a^
Peroneus brevis	1.26 ± 0.39	0.61 ± 0.08	[0.37, 0.93]	1.92	0.000^a^	1.63 ± 0.99	0.57 ± 0.14	[0.34, 1.77]	1.17	0.008^a^
Flexor digitorum longus	0.97 ± 0.19	0.82 ± 0.40	[−0.14, 0.45]	0.11	0.29	0.92 ± 0.24	0.75 ± 0.25	[−0.06, 0.39]	0.53	0.143
Posterior tibial	1.06 ± 0.32	0.81 ± 0.22	[−0.01, 0.51]	0.42	0.055	0.85 ± 0.14	0.83 ± 0.19	[−0.14, 0.18]	0.44	0.776
Flexor hallucis longus	1.10 ± 0.36	0.74 ± 0.39	[0.02, 0.72]	0.19	0.041^a^	1.06 ± 0.29	0.78 ± 0.34	[−0.01, 0.58]	0.06	0.057
Triceps surae	1.23 ± 0.58	0.80 ± 0.43	[−0.06, 0.91]	0.32	0.079	1.16 ± 0.54	0.73 ± 0.25	[0.03, 0.83]	0.54	0.035^a^
Abductor hallucis	1.99 ± 1.33	0.74 ± 0.24	[0.29, 2.20]	1.01	0.016^a^	1.30 ± 0.50	0.74 ± 0.34	[0.16, 0.96]	0.97	0.009^a^
Plantar quadrate	1.19 ± 0.52	0.77 ± 0.16	[0.05, 0.78]	0.67	0.028^a^	1.16 ± 0.44	0.82 ± 0.23	[0.01, 0.68]	0.54	0.043^a^
Flexor digitorum brevis	1.55 ± 0.76	0.79 ± 0.21	[0.21, 1.31]	1.06	0.012^a^	1.44 ± 0.76	0.71 ± 0.22	[0.18, 1.28]	1	0.015^a^
Abductor digiti minimi	1.13 ± 0.44	0.69 ± 0.27	[0.10, 0.79]	1.08	0.014^a^	1.34 ± 0.68	0.69 ± 0.24	[0.18, 1.13]	0.91	0.010^a^
Flexor hallucis brevis	1.75 ± 0.68	0.77 ± 0.26	[0.47, 1.49]	1.71	0.001^a^	1.81 ± 0.66	0.81 ± 0.31	[0.50, 1.51]	1.65	0.001^a^
Abductor hallucis	1.65 ± 0.91	0.80 ± 0.24	[0.19, 1.51]	1.09	0.017^a^	1.58 ± 0.54	0.87 ± 0.33	[0.28, 1.12]	1.43	0.003^a^
Interosseous	1.37 ± 0.59	0.68 ± 0.22	[0.25, 1.12]	1.38	0.005^a^	1.21 ± 0.49	0.71 ± 0.23	[0.13, 0.88]	1.3	0.012^a^

^a^Statistically significant

*SUV* Standardized uptake value

Values are presented as mean ± standard deviation

## Discussion

In this study, the whole-body skeletal muscle metabolism during pitching motion was evaluated using PET. An accumulation of FDG was observed in the relatively small skeletal muscles of the fingers and toes, and asymmetric accumulation was observed in the thigh muscles. Interestingly, there was minimal accumulation of FDG in the rotator cuff and trunk muscles, which are considered important for the throwing motion.

EMG has been used in the evaluation of skeletal muscle activity during pitching [1, 6–13]. Since EMG has excellent time resolution, it was useful for investigating skeletal muscle activity in each throwing phase by synchronizing the time axis to motion analysis [4]. However, when using EMG with surface electrodes, since it is necessary to attach the electrode beforehand to the muscle to be studied, it is only possible to measure the muscle that can be touched from the body surface and the muscles scheduled for investigation [4–6]. Therefore, it is difficult to observe the influence of exercise on skeletal muscle activity in deep-lying regions of the trunk and extremities [16]. Alternatively, wire electrodes can be used for deeper skeletal muscles; however, they are invasive [8, 12]. In addition, the accuracy of the insertion site and the influence of noise artifacts similarly have a significant impact [8, 12]. Furthermore, in evaluation using EMG, the skeletal muscles of the whole-body are invisible, and the device’s cords and electrodes interfere with motion [16]. Hence, it becomes impossible to study muscle activity in conditions that actually replicate the real-world pitching environment and performance [16]. Therefore, only partial investigations on the upper limbs [6–9, 11, 12], lower limbs [4, 10, 13], or the trunk [14] have been performed in pitchers so far.

Some studies have utilized FDG-PET to display cumulative muscle metabolism during exercise [15, 16, 18–20]. FDG taken up by muscle cells is not metabolized and remains in the cells as FDG-6-phosphate after phosphorylation [15]. Thus, FDG accumulation in the muscle can be used as a parameter of glucose uptake by the muscle and is an indicator of muscle activity [15]. Glucose metabolism measured by FDG-PET demonstrates a high correlation with the intensity of muscle activity, and its reliability as an index for measuring muscle activity has been confirmed [20]. Fujimoto et al. [19] and Tashiro et al. [21] used PET in the evaluation of muscle metabolism during running (one of the first studies). Other studies have investigated tissue glucose uptake with PET during tasks such as isometric muscle contractions [15] and dynamic strength exercises [22], and during more complex tasks requiring endurance, such as walking [23], running [24], and double poling [25]. In a previous study, whole-body PET-CT was used to examine where glucose uptake occurs in the skeletal muscles in a single performance of Part 2 of the Fédération Internationale de Football Association’s 11+ injury prevention program [15] and following its 4-week routine performance [16]. Bojsen-Møller et al. [25] proposed that PET imaging might be a promising adjunct modality or an alternative to more traditional methods of investigating muscle activity during complex human movements. Rudroff et al. [22] mentioned that PET has the potential of being the “gold standard” of metabolic imaging for exercise physiology.

In this study, it was observed that the accumulation of FDG in the relatively small skeletal muscles of the fingers, especially in the muscles that have their origin and insertion within the hand (intrinsic muscles). The fingers control, which is the end of the kinetic chain of the pitching motion, is extremely precise and important [26]. The intrinsic muscles are known to play a vital role in the flexing and stabilization of the metacarpophalangeal (MCP) joint. Ketchum et al. [27] reported that the intrinsic muscles contribute more than 70% of the overall moment of the flexion at the MCP joint. However, studies on the intrinsic finger muscles activity during pitching have not been conducted. This is presumably because the evaluation of intrinsic muscles is difficult with EMG. Using another approach, Kinoshita et al. [28] measured finger force for fastball pitching; a linear relationship was observed between peak forces and ball velocity, and the peak ball reaction force for fastballs exceeded 80% of maximum finger strength. According to these studies, the training of finger muscles is important for enhancing pitching performance [26–28]. Similarly, FDG accumulation increased in multiple intrinsic foot muscles in this study. The control of the intrinsic foot muscles is equally important for the first step in the kinetic chain of the pitching motion [29]. However, the intrinsic foot muscles activity has not attracted significant attention until now due to the difficulty to be evaluated with EMG [4, 10].

Some skeletal muscles of the femoral region demonstrated unilateral accumulation of FDG. This might reflect the asymmetric movements performed during pitching. The pitching motion has been classified into six phases [30]. Specifically, it was suggested that the iliacus on the non-throwing side is involved in the lifting movement of the lower limbs during the wind-up phase [4, 10, 13, 29, 30]. On the contrary, the medial hamstrings, vastus medialis, and adductor muscle on the throwing side are responsible for the translational motion during the stride phase to the acceleration phase and the raising motion of the lower extremity during the deceleration phase [4, 10, 13, 29, 30]. The sartorius and gracilis muscles on the non-throwing side are involved in the process of standing up on one leg during the acceleration to follow-through phase [4, 10, 13, 29, 30]. In a previous EMG study, Yamanouchi [10] investigated the abductor, adductor, quadriceps, biceps, tibialis anterior, and gastrocnemius muscles activities; it was concluded that the abductor and adductor muscles play important roles. Similarly, activation of the adductor muscle was observed in this study. In the investigation of the vastus medialis, biceps femoris, rectus femoris, gluteus maximus, and gastrocnemius muscles activities by Campbell et al. [4], it was concluded that stride foot contact on ball release was the most demanding phase of the pitching motion with a very high bilateral muscular activation. The iliacus and sartorius muscles were excluded from the investigation [4]. Since the iliac muscle is in a deep position, it would be difficult to investigate its activity using EMG. Regarding the biceps femoris muscle on the throwing side, reports equally indicated a high activity [4, 10, 13]. In addition, Erickson et al. [13] conducted EMG studies and reported a higher hamstring activity in the throwing side than the non-throwing side, which is consistent with this study.

Interestingly, minimal accumulation of FDG was observed in the rotator cuff and trunk muscles in this study, suggesting the possibility that these muscles contribute less to the throwing movement. The shoulder is an integral part of the kinetic chain in the throwing motion. Rotator cuff muscles act as dynamic stabilizers of the glenohumeral joint during throwing [6, 7]. An initial report of shoulder muscle activity during baseball pitching evaluated by EMG was published in 1983 by Jobe et al. [11]. Since then, considerable research using EMG for the muscles around the shoulder joint during pitching has been performed [6–9, 11, 12]. According to these studies, the rotator cuff demonstrated high activity during the early cocking to deceleration phases [6–9, 11, 12]. This study showed no increase in glucose metabolism in the rotator cuffs. Around the shoulder joint, increased metabolism was observed in the posterior aspect of the deltoid muscles on both sides, the trapezius muscle, teres major, latissimus dorsi on the pitching side, and the levator scapulae on the non-throwing side in this study. Similarly, increased metabolism in both sides of the biceps and brachial muscles was observed in this study.

There is only one report on muscle activity of the trunk during pitching measured by EMG [14]. In the investigation of the muscle activity of the abdominal rectus, abdominal oblique, lumbar paraspinous, and gluteus maximus muscles by Watkins et al. [14], it was reported that the abdominal rectus and abdominal oblique muscles on the non-throwing side demonstrated an increase in the activity level. In this study, no increase in glucose metabolism was observed in the trunk muscles; however, increased metabolism was observed only in the gluteus maximus muscle on the throwing side.

Understanding the patterns of whole-body muscle metabolism in baseball pitching is important to enhance performance and decrease injury potential [4]. For practical application, data from this study provide strength and conditioning professionals more definitive evidence on the importance of finger and foot muscular strengths and endurance training for pitchers. Furthermore, training regimens promoting both bilateral and unilateral lower extremity muscular strengths and endurance in multiple planes (similar to the movements in pitching) are critical to address the specific demands of the pitching motion [1, 3, 4, 30, 31].

This study had some limitations. First, the number of throws that is optimal for measuring FDG accumulation is unclear. Based on previous studies, it was better to exercise for 20 min before and after the FDG injection. Thus, the participants threw 40 balls in 20 min before and after FDG injection. However, since the pitching motion was completed in approximately 2 s, the appropriateness of the exercise load in this study is questionable. In addition, it is possible that the motion and the pattern of muscle metabolism changed with fatigue [13]. We are planning to examine these issues by increasing the number of throws in future studies. Second, the method of FDG-PET only accounted for muscle glucose uptake. Other substrates, such as free fatty acids, muscle glycogen, and lactate are metabolized in active muscle cells. Nonetheless, glucose oxidation increases with exercise intensity, and glucose uptake increases fairly, in proportion to glycogen utilization with an increase in the intensity of exercise [31]. Third, the method used to define ROI. As FDG uptake was measured at an arbitrary site on the target muscle, it did not reflect the uptake of the entire muscle volume; therefore, further investigation of muscle metabolism using PET is required. Fourth, the sample size was limited due to concerns over radiation exposure. The effect size of the mean SUV of the peroneus brevis was 2.33 on the throwing side, which was greater than the expected level of 2.0; this was the basis for calculating the sample size before the investigation. Thus, the sample size of the pitcher and control groups (*n* = 10 each) in this investigation was appropriate. Despite these limitations, no study has employed PET-CT in the examination of the whole-body muscle metabolism (during pitching).

To the best of our knowledge, this is the first study to evaluate whole-body skeletal muscle metabolism during pitching. PET is a useful tool for measuring the skeletal muscle metabolism of the whole body in an environment that accurately replicates actual pitching and is, therefore, useful for assessing other dynamic activities. As EMG research can only measure specific parts of the body, it was necessary to integrate several different studies of various participants and methods to consider and devise a novel whole-body training method.

## Conclusions

Whole-body muscle metabolism during pitching was investigated using PET-CT, and a significant increase in glucose metabolism was observed in muscle groups of the fingers and toes. In addition, asymmetric metabolism was observed in the thigh muscles. There was minimal accumulation of FDG in the rotator cuff and trunk muscles. This information would be useful in determining the training required for pitchers. These findings can be equally applied to other sporting activities that involve throwing.

## Data Availability

The datasets generated during and/or analyzed during the current study are available from the corresponding author on reasonable request.

## Abbreviations

3D: 3-dimensional
CT: Computed tomography
EMG: Electromyography
FDG: ^18^F-fluorodeoxyglucose
MCP: Metacarpophalangeal
PET: Positron emission tomography
ROI: Regions of interest
SD: Standard deviation
SUV: Standardized uptake value
